# Reduction in Serum Carotenoid Levels Following One Anastomosis Gastric Bypass

**DOI:** 10.3390/nu16162596

**Published:** 2024-08-07

**Authors:** Ayelet Harari, Osnat Kaniel, Rom Keshet, Aviv Shaish, Yafit Kessler, Amir Szold, Peter Langer, Asnat Raziel, Nasser Sakran, David Goitein, Jacob Moran-Gilad, Shiri Sherf-Dagan

**Affiliations:** 1 The Bert W. Strassburger Lipid Center, Sheba Medical Center, Tel-Hashomer 5262000, Israel; ayelet.harari@sheba.health.gov.il (A.H.); rom.keshet@sheba.health.gov.il (R.K.); aviv.shaish@sheba.health.gov.il (A.S.); 2 Disaster Readiness Management Unit, Sanz Medical Center, Laniado Hospital, Netanya 4244916, Israel; osnatyy@gmail.com; 3 Achva Academic College, Beer-Tuvia Regional Council 7980400, Israel; 4 Department of Nutrition Sciences, School of Health Sciences, Ariel University, Ariel 40700, Israel; yafitke@gmail.com; 5 Assia Medical Group, Assuta Medical Center, Tel-Aviv 6971028, Israel; amikisz@gmail.com (A.S.); pplanger@gmail.com (P.L.); doctor@asnatraziel.com (A.R.); sakranas@gmail.com (N.S.); david.goitein@sheba.health.gov.il (D.G.); 6 Assuta Bariatric Centers, Assuta Hospitals, Tel-Aviv 6971028, Israel; 7 Division of General Surgery, Sheba Medical Center, Tel-Hashomer 5262000, Israel; 8 Faculty of Medicine, Tel-Aviv University, Tel-Aviv 6997801, Israel; 9 Department of Surgery, Holy Family Hospital, Nazareth 1601001, Israel; 10 The Azrieli Faculty of Medicine Safed, Bar-Ilan University, Ramat Gan 5290002, Israel; 11 Department of Health Policy and Management, School of Public Health, Faculty of Health Sciences, Ben-Gurion University of the Negev, Beer-Sheva 8410501, Israel; giladko@post.bgu.ac.il; 12 Department of Nutrition, Assuta Medical Center, Tel-Aviv 6971028, Israel

**Keywords:** carotenoids, dietary intake, metabolic bariatric surgery, one anastomosis gastric bypass

## Abstract

Given the health benefits of carotenoids, it is crucial to evaluate their levels in patients undergoing malabsorptive procedures like one anastomosis gastric bypass (OAGB). This study aimed to assess serum carotenoid levels before and 6 months following OAGB. Prospectively collected data from patients who underwent primary OAGB were analyzed. Data included anthropometrics, dietary intake assessments, and biochemical tests. Serum samples were analyzed for lipid profile and serum carotenoids, including lutein, zeaxanthin, α-carotene, β-carotene, phytofluene, ζ-carotene, and lycopene. Data from 27 patients (median age 47.0 years and 55.6% female) were available before and 6 months post-OAGB. The median pre-surgical BMI was 39.5 kg/m^2^, and the median excess weight loss at 6 months post-surgery was 63.9%. Significant decreases in all carotenoid levels were observed over time (*p* < 0.001 for all). A median relative decline of 65.1% in absolute total carotenoid levels and 12.7% in total cholesterol levels were found. No associations were observed between changes in clinical outcomes and carotenoid levels during the study period. This study reveals significant decreases in carotenoid levels within the first 6 months following OAGB. Nutritional intervention studies are needed to explore how incorporating carotenoid-rich foods affects post-surgery carotenoid levels and clinical outcomes.

## 1. Introduction

Metabolic bariatric surgery (MBS) is an effective treatment modality for severe obesity [1,2]. Several procedure types are available, with each having pros and cons [3]. One anastomosis gastric bypass (OAGB) is an emerging bariatric procedure in which a long narrow sleeve gastric tube in conjunction with a side-to-side or end-to-side gastro jejunostomy performed generally 150–200 cm distal to the Treitz’ ligament [4,5,6]. In Israel, a significant increase in OAGB performance became evident in recent years (in 2015, 7.4%; in 2016,18.5%; in 2017, 32.7%; in 2018, 46.4%; in 2019, 56.2%, in 2020, 63.7%, and in 2021, 68.2%) [7,8]. Globally, OAGB constituted 6.6% of MBS procedures in 2018 and 7.6% in 2021 [9].

At present, the nutritional implications of OAGB are not fully understood [10]. While studies have presented information on nutritional deficiencies and malnutrition following OAGB [11,12,13,14,15,16,17,18,19,20,21], data regarding the effect of the surgery on phytochemical levels are missing.

Carotenoids, natural pigments produced by algae, plants, bacteria, and fungi, varying in color from yellow to red, are predominantly sourced from vegetables and fruits in the diet as they cannot be synthesized within the human body [22,23,24,25]. Carotenoids are transported along with other lipids in plasma lipoproteins, primarily LDL and, to a lesser extent, HDL [26]. Of over 1000 known carotenoids, only around 50 are present in our diets, and of those, only about a dozen are detectable in the human bloodstream and carried by lipoproteins [27]. Dietary carotenoids can be divided into provitamin A carotenoids (e.g., β-carotene, α-carotene, and β-cryptoxanthin) and non-provitamin A carotenoids (e.g., lycopene, lutein, and zeaxanthin) [23,24]. Beyond their role as provitamin A precursors, carotenoids exhibit antibacterial, immunological, and anti-inflammatory properties [28,29]. High dietary carotenoid consumption and their concentrations in various body compartments have been linked to a decreased incidence of several chronic diseases and premature mortality [23,30]. High plasma carotenoid levels are associated with reduced risks of certain types of cancers; type 2 diabetes; cardiovascular disease; and bone, skin, or eye disorders [23,30,31,32]. Conversely, obesity and overweight are correlated with lower blood carotenoid levels, and serum carotenoids are inversely correlated with total body fat mass and central fat mass [31]. Nevertheless, the exact mechanism for reduced carotenoid levels in obesity remains uncertain and might indicate a poor-quality diet that lacks sufficient carotenoid intake [33].

Assessing serum carotenoid levels before and after MBS, especially malabsorptive procedures like OAGB, is crucial for developing specific dietary recommendations for these patients. Nevertheless, this issue has received minimal attention thus far [22,34].

This study aims to evaluate serum carotenoid levels before and 6 months following OAGB and to investigate the associations between alternation in serum carotenoid levels and changes in anthropometric and nutritional measures over the study period. We hypothesized that serum carotenoid levels would significantly change post-OAGB and that these changes would be associated with improvements in anthropometric and nutritional measures.

## 2. Methods

### 2.1. Study Participants

A retrospective analysis was conducted on data collected prospectively (i.e., pre-surgery and at 6 months post-surgery) among 32 patients who underwent primary OAGB at the Assuta Medical Centers between 2018 and 2019 [35]. Inclusion criteria of the original prospective study based on age (i.e., pre-surgery age of 18–65 years), pre-surgery body mass index (BMI) of >40 kg/m² or BMI > 35 kg/m² with medical-associated conditions, and having the approval of the Assuta Medical Centers committee to undergo primary MBS. Exclusion criteria included usage of antibiotics or probiotics in the month preceding the MBS; using promotility drugs and laxatives one week before each study examination day; having an uncontrolled mental illness, cognitive deterioration, or chronic conditions that could interfere with the study; treatment with insulin; excessive alcohol consumption; and present pregnant or breastfeeding [35]. Patients received standard treatment recommendations during the study period [36,37].

The study protocol was approved by the ethics committee of the institutional review board of Assuta Medical Centers (#0014-18-ASMC and #0046-23-ASMC).

### 2.2. Pre- and Post-Surgical Follow-Up Measurements

In the original prospective study [35], pre-surgical and follow-up assessments at 6 months post-surgery encompassed a wide range of evaluations. These included gut microbiome analysis, glucose breath tests, biochemical tests, anthropometrics, dietary intake assessments, questionnaires to evaluate gastrointestinal symptoms and quality of life, and interviews to assess medical status and lifestyle habits.

#### 2.2.1. Anthropometrics

Height was assessed without shoes using a wall-mounted altimeter, weight was measured using a high-capacity weigh scale, and BMI was then calculated. Excess weight loss (%EWL) and total weight loss (%TWL) percentages were computed according to established recommendations [38]. Also, waist circumference (WC) was measured twice at the umbilicus level, and the mean was calculated [39].

#### 2.2.2. Dietary Intake Assessments

Dietary intake the month before testing was assessed using a Food Frequency Questionnaire (FFQ), as previously described [40]. Energy and macronutrient intake for the food items were obtained from the Israeli nutritional software ‘Zameret’ version 4 [41].

#### 2.2.3. Biochemical Tests

Blood tests were conducted following a 12 h fast, encompassing blood count, as well as transferrin, ferritin, iron, vitamin B12, folic acid, vitamin A, and vitamin D levels. Nutritional abnormalities were operationally defined as plasma levels falling below or above the recommended reference range provided by the laboratory or clinical practice [42]. Frozen serum samples from all participants were immediately stored at −80 °C until analyses were conducted for lipid profile and serum carotenoid analysis. High-performance liquid chromatography (HPLC( detected several carotenoids and their stereoisomers, including lutein, zeaxanthin, α-carotene, β-carotene, phytofluene, ζ-carotene, and lycopene. Carotenoid levels are reported as absolute concentrations and adjusted to total cholesterol levels, as carotenoids are carried by lipoproteins in the bloodstream [43].

#### 2.2.4. Carotenoid Analysis

Serum (0.5 mL) was extracted with 2 mL ethanol containing 10 µM butylated hydroxytoluene. After the addition of 2 mL hexane and 1 mL double-distilled water (DDW), the samples were mixed and centrifuged for 5 min at 1000× *g* at 4 °C. The hexane layers of the serum were dried in N_2_. Dried samples were then suspended in 100 µL tert butyl methyl ether. Serum carotenoids were determined by reverse-phase HPLC on a YMC C30 column (CT995031546QT, 150 × 4.6, 3-μm particle size; YMC, Inc. Kyoto, Japan) with a gradient, as previously described [44]. Carotenoids were detected by monitoring absorbance and through a comparison with the retention times of authentic standards (95–98% purity; CaroteNature, Switzerland). Absorbance was detected at a wavelength of 200 to 700 nm to identify carotenoids with absorption in the UV and visible range of the spectrum.

### 2.3. Statistical Methods

The SPSS statistical package Version 29 was used for all statistical analyses. Continuous variables are presented as median and interquartile range (IQR) and dichotomous/categorical variables as proportions. The Spearman correlation coefficient was used to test the correlations between continuous variables. Continuous variables between two time points were compared using the Wilcoxon signed-rank test, while in dichotomous or categorical variables the McNemar test was used. The level of significance for all analyses was set at *p* < 0.05.

#### Power Calculation

When applying a sample size of N = 27, a 0.05 two-sided alpha level, large effect size (Cohen d = 0.8) [45] in G*power software version 3.1.9.7 to test differences between two dependent means, a power of >0.9 was calculated.

## 3. Results

### 3.1. Characteristics of the Study Population

Among the thirty-two patients included in our baseline analyses, one canceled the surgery, one underwent MBS in another hospital, one withdrew from the study, and two lacked serum samples for carotenoid analysis. Therefore, data on 27 patients, with a median age of 47.0 (ranged 18.0 to 62.0) years and 55.6% (n = 15) female, were available for this analysis before and 6 months post-OAGB. Among these patients, 11.1% (n = 3) had type 2 diabetes, 59.3% (n = 16) had dyslipidemia, and 22.2% (n = 6) had hypertension. The median length of the bypassed limb conducted in the surgery was 180 cm (ranged 150 to 200 cm). Anthropometrics and nutritional characteristics of the study participants at baseline and 6 months post-surgery are presented in Table 1. The median pre-surgical BMI was 39.5 kg/m^2^, and at 6 months post-surgery, the median EWL was 63.9% (ranged 38.5 to 118.3%). Energy and macronutrient intake decreased significantly between baseline and 6 months post-OAGB (*p* < 0.001 for all). Multivitamin intake was reported by 44.4% and 92.6% at baseline and 6 months post-OAGB (*p* < 0.001), respectively.

### 3.2. Changes in Lipoprotein Levels during the Study Period

Median and IQR total cholesterol levels at baseline and 6 months post-OAGB were 181.0 mg/dL (165.0, 209.0) and 162.0 mg/dL (144.0, 183.0), respectively, indicating a median relative decrease of 12.7%. Median and IQR LDL levels at baseline and 6 months post-OAGB were 113.0 mg/dL (99.0, 135.0) and 99.0 mg/dL (81.0, 108.0), respectively, indicating a median relative decrease of 22.0%. Median and IQR HDL levels at baseline and 6 months post-OAGB were 47.0 mg/dL (38.0, 57.0) and 48.0 mg/dL (44.0, 55.0), respectively, indicating a median relative increase of 4.7%. Median and IQR VLDL levels at baseline and 6 months post-OAGB were 26.0 mg/dL (20.0, 33.0) and 19.0 mg/dL (16.0, 22.0), respectively, indicating a median relative decrease of 24.1%. Median and IQR non-HDL levels at baseline and 6 months post-OAGB were 136.0 mg/dL (119.0, 169.0) and 115.0 mg/dL (96.0, 128.0), respectively, indicating a median relative decrease of 22.9%. Median and IQR triglyceride levels at baseline and 6 months post-OAGB were 128.0 mg/dL (100.0, 166.0) and 93.0 mg/dL (81.0, 111.0), respectively, indicating a median relative decrease of 24.5%.

### 3.3. Changes in Carotenoid Levels during the Study Period

**Figure 1:**
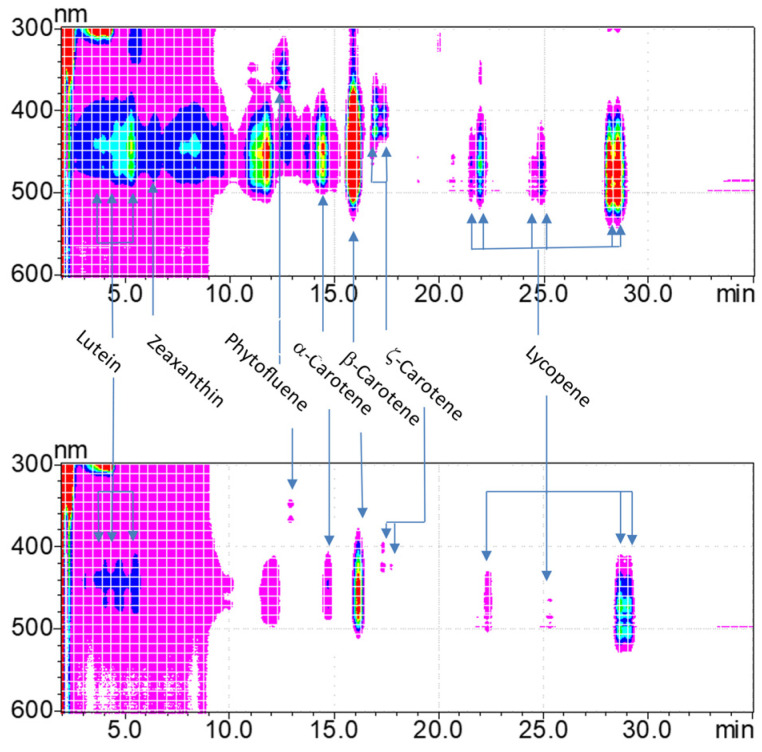
A representative HPLC^1^ analysis spectra of serum carotenoids before and 6 months post-OAGB (spectrum range 200–700 nm). ^1^ High-performance liquid chromatography (HPLC). The pink and the red colors represent the lowest and the highest absorbance in the contour plot, respectively.

Overall, one isomer of phytofluene, α-carotene, and β-carotene, three isomers of lutein and ζ-carotene, and six isomers of lycopene were identified. Nonetheless, according to absorption spectra, additional isomers of these carotenoids may have also been present, but their identification could not be confirmed or was below the limit of quantification (Figure 1).

**Figure 2:**
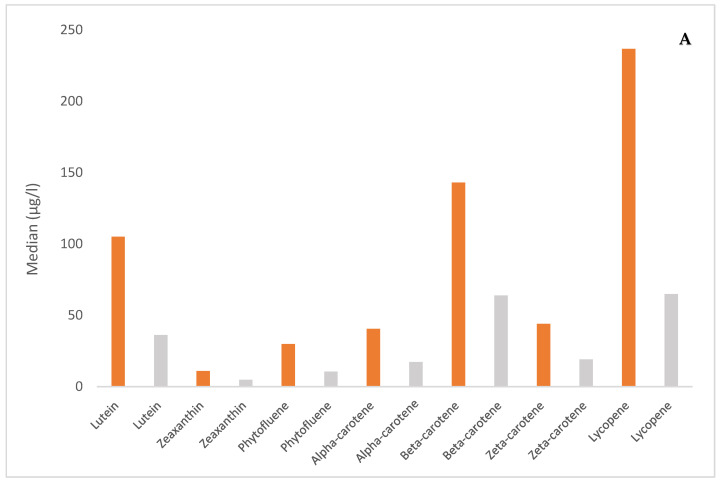

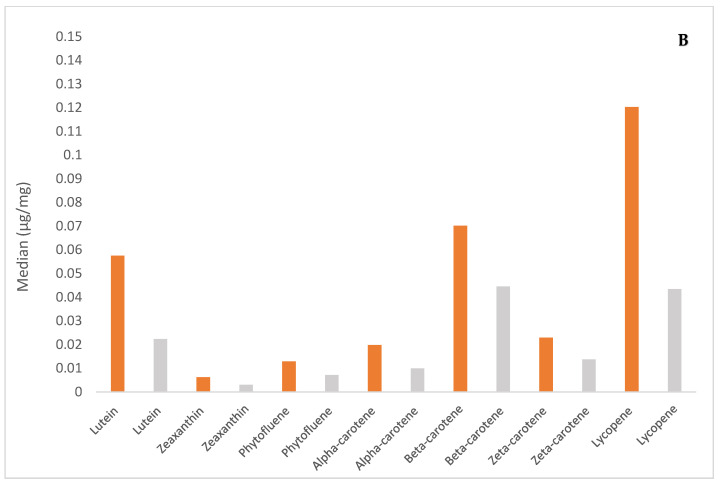
(**A**) Absolute carotenoid levels before and 6 months post-OAGB (N = 27). All paired comparisons were found statistically significant (*p* < 0.001). (**B**) Adjusted carotenoid to total cholesterol levels before and 6 months post-OAGB (N = 27). All paired comparisons were found statistically significant (*p* ≤ 0.003). Baseline results are shown in orange, and 6 months post-OAGB results are shown in gray.

A significant decrease in all absolute carotenoid levels and adjusted carotenoid to total cholesterol levels was observed between baseline and 6 months following OAGB (*p* < 0.001 for all and *p* ≤ 0.003, respectively) (Figure 2A,B and Supplementary Table S1). The median and IQR levels of total carotenoid levels at baseline and 6 months post-OAGB were 618.4 µg/L (528.9, 832.2) and 216.3 µg/L (183.5, 303.6), respectively (*p* < 0.001). The median and IQR levels of total carotenoid adjusted to total cholesterol levels at baseline and 6 months post-OAGB were 0.366 µg/mg (0.264, 0.459) and 0.137 µg/mg (0.111, 0.196), respectively (*p* < 0.001).

**Figure 3:**
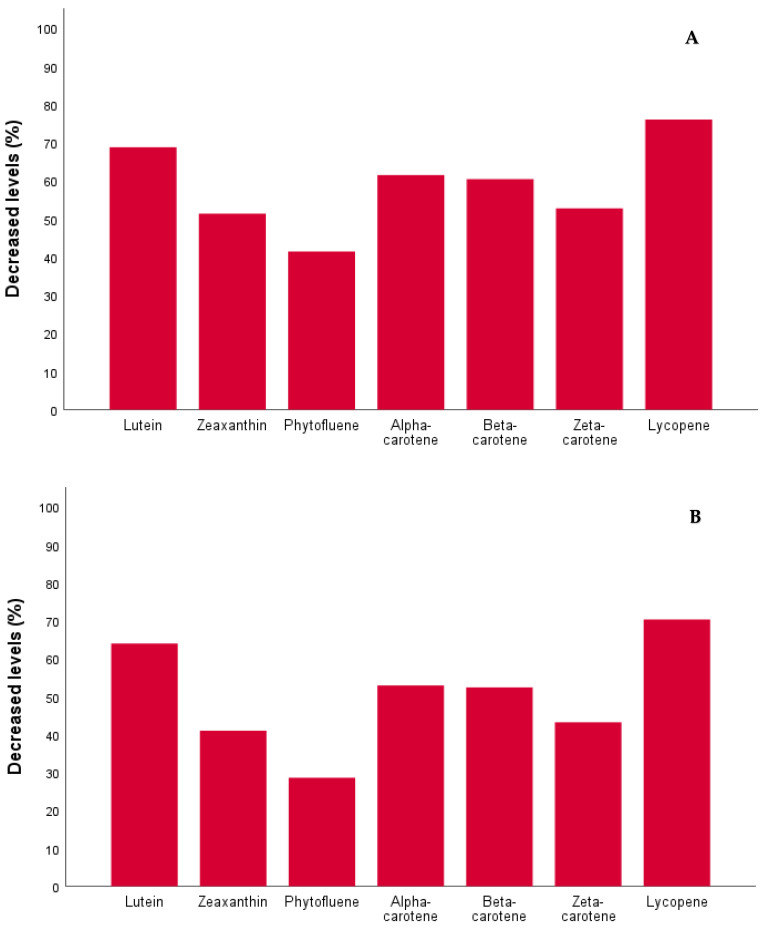
(**A**) Decrease in percentages of absolute carotenoid levels at 6 months post-OAGB compared to baseline (N = 27). (**B**) Decrease in percentages of adjusted carotenoid to total cholesterol levels at 6 months post-OAGB compared to baseline (N = 27).

A significant proportion of patients exhibited a downward trend in absolute levels from baseline for lutein (92.6%), zeaxanthin (92.6%), α-carotene (96.3%), β-carotene (92.6%), ζ-carotene (92.6%), phytofluene (85.2%), lycopene (100%), and total carotenoids (100%). The relative decrease in absolute carotenoid levels at 6 months post-OAGB compared to baseline is depicted in Figure 3A. At 6 months post-OAGB, the median of absolute total carotenoid levels decreased by 65.1% compared to baseline.

A significant proportion of patients exhibited a downward trend in carotenoid levels adjusted for total cholesterol, with an absolute decrease from baseline observed in lutein (92.6%), zeaxanthin (85.2%), α-carotene (92.6%), β-carotene (92.6%), ζ-carotene (81.5%), phytofluene (77.8%), lycopene (100%), and total carotenoids (96.3%). The relative decrease in adjusted carotenoid to total cholesterol levels at 6 months post-OAGB compared to baseline is depicted in Figure 3B. At 6 months post-OAGB, the median adjusted total carotenoid to total cholesterol levels decreased by 61.4% compared to baseline.

Although a significant reduction in absolute carotenoid levels was observed at a group level during the study period, some variability between patients was found (Supplementary Figure S1).

### 3.4. Associations between Changes in Carotenoid Levels and Clinical Parameters during the Study Period

No associations were observed between changes in anthropometrics, caloric intake, macronutrient intake, and changes in absolute carotenoid levels or carotenoid levels adjusted for total cholesterol during the study period.

## 4. Discussion

The growing understanding of the beneficial role of carotenoids in health outcomes [30,31,32] mandates evaluation among patients undergoing MBS, especially malabsorptive procedures like OAGB.

In the present study, significant decreases in carotenoid levels were observed 6 months following OAGB. Additionally, the reductions in carotenoid levels were not related to changes in anthropometric parameters, energy, or macronutrient intake.

A possible explanation for the decrease in carotenoid levels post-OAGB may include interference with fat absorption induced by this surgery, as carotenoids are fat-soluble phytochemicals [24]. The reduced levels of serum carotenoids may also be attributed to lower circulating concentrations of lipoproteins, which function as carotenoid transporters in the bloodstream [22,43]. Nonetheless, although the lipoproteins showed significant decreases during the study period, the reduction in carotenoid levels was about three-fold higher. Additionally, lipid-adjusted concentrations demonstrated a clear decreasing trend over time as absolute concentrations. Also, inadequate intake of carotenoid-rich foods following the surgery may affect their serum decline over time [34]. Nevertheless, it is worth mentioning that although a correlation between carotenoid intake and blood and tissue levels exists, various host factors can influence bioaccessibility and bioavailability [23]. Some multivitamin products targeted for MBS populations include synthetic β-carotene in multiple forms and doses but no other carotenoids. Even though most participants in this study consume multivitamins post-OAGB, the effect on β-carotene levels seemed minimal, as the decrease in this carotenoid was similar to others over time. This notable reduction in β-carotene levels over time may be due to its conversion to vitamin A, given that β-carotene is a provitamin A carotenoid [23,24]. In the present study, at 6 months following OAGB, vitamin A levels were in the normal range for most patients, with only 15% of participants presenting a deficiency. Additionally, vitamin D levels remained stable 6 months post-OAGB [35]. While long-term suitable supplementation post-surgery is the key pillar of MBS [36], adherence tends to decrease with time [10,46], and absorption of all elements is not always guaranteed. Caution should be taken when administering high doses of synthetic β-carotene supplementation alone or in combination with other antioxidants, especially to at-risk populations such as smokers [47,48].

Data on the prevalence and implications of carotenoid levels following MBS are scarce [22,34]. In a retrospective cohort study involving 23 participants before and 53 participants on average 18 months following Roux-en-Y gastric bypass (RYGB) (n = 20) and 14 months following biliopancreatic diversion (BPD) (n = 33), all carotenoid levels significantly dropped post-surgery even after adjustment for cholesterol levels, particularly among those who underwent BPD [34]. A follow-up study that included 75 participants before and 362 participants on average 15 months following RYGB (n = 187) and 18 months following BPD (n = 175) also presented a continuous drop of all carotenoid levels over time [22]. These trends align with our results. Additionally, in the present study, we measured phytofluene and ζ-carotene, which were found in higher proportions in human adipose tissue compared to serum [31]. However, cryptoxanthin was not measured in this study.

**Figure 4:**
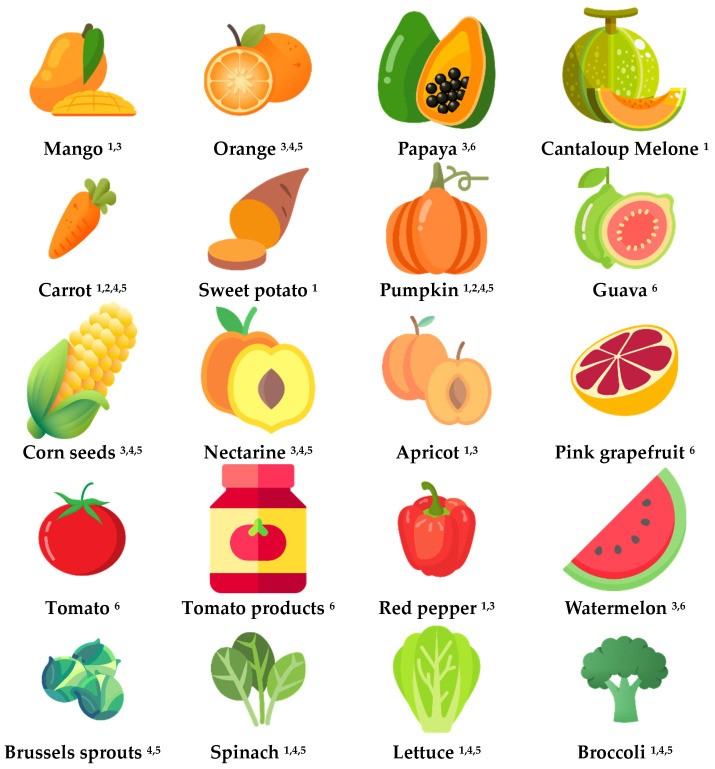
Various plant-based products are known for being rich sources of carotenoids *. ^1^ rich in β-carotene; ^2^ rich in α-carotene; ^3^ rich in β-cryptoxanthin; ^4^ rich in lutein; ^5^ rich in zeaxanthin; ^6^ rich in lycopene. * Icons made by www.flaticon.com [credits can be found here], accessed on 15 May 2024.

Currently, no dietary reference intakes for carotenoids exist, and intake suggestions are primarily derived from epidemiological studies [23,24]. Carotenoids are consumed through direct ingestion of fruits and vegetables, and through artificial sources such as carotenoid supplements or added food colorants [23,24]. Therefore, as serum levels depend predominantly on their content in the diet, a rational approach could be to offer dietary advice on incorporating carotenoid-rich and fortified foods into the diet which could be beneficial for patients undergoing malabsorptive MBS, specifically OAGB. Figure 4 illustrates a variety of plant-based products known for being rich sources of carotenoids [24,25,49].

The strengths of the current study include the various carotenoid types tested before and 6 months post-OAGB, along with other clinical parameters. Nevertheless, several limitations should be noted. First, the sample size was relatively small, thus limiting the statistical power. Nonetheless, we found a significant difference despite the sample size. Second, data on body composition, carotenoid intake, level of other fat-soluble vitamins, and inflammatory markers were unavailable in the present study. Although the liver is the main organ that stores carotenoids, significant amounts also accumulate in adipose tissue [31,50]. Therefore, the substantial loss of adipose tissue mass following MBS could be reflected in serum carotenoid levels [22]. Third, normal and deficiency threshold range concentrations for carotenoids have yet not been established [23]. Thus, our findings reflect a notable reduction in values, not necessarily a deficiency. Carotenoids are crucial for preventing chronic diseases because of their antioxidant, anti-inflammatory, and immunoprotective properties [28]. Consequently, a significant decrease in carotenoid levels following MBS could increase the long-term risk of chronic diseases.

## 5. Conclusions

Our study reveals significant decreases in absolute carotenoid and carotenoid adjusted for total cholesterol levels within the first 6 months following OAGB. Notably, these decreases appear to be independent of changes in anthropometric parameters, energy, or macronutrient consumption. Future large-scale prospective cohort studies should examine changes in carotenoid levels before and after different types of MBS and explore the risk factors associated with developing decreased levels over time and their clinical implications. Additionally, there is a need for nutritional intervention studies to examine the effect of incorporating carotenoid-rich food into the post-surgery diet on serum carotenoid levels and other clinical outcomes.

## Figures and Tables

**Table 1:** Anthropometrics and nutritional characteristics of the study participants at baseline and 6 months post-surgery (N = 27).

Variable ^1^	Baseline	6 Months Post-Surgery	*p* Value
**Anthropometrics**			
Height (meter)	1.68 (1.62, 1.80)	-	-
Weight (kg)	110.6 (104.6, 130.4)	86.4 (77.4, 101.4)	<0.001
BMI (kg/m²)	39.5 (37.1, 47.2)	31.0 (27.3, 34.0)	<0.001
WC (cm)	118.0 (113.0, 127.0)	97 (89.0, 110.0)	<0.001
%EWL		63.9 (48.4, 81.3)	-
**Dietary intake**			
Calories (kcal/day)	2383 (1920, 3219)	1584 (1024, 2136)	<0.001
Protein (g/day)	123 (103, 151)	84 (54, 113)	<0.001
Carbohydrates (g/day)	209 (164, 275)	130 (108, 205)	<0.001
Fats (g/day)	99 (83, 137)	68 (45, 84)	<0.001
Taking multivitamins (%yes)	44.4	92.6	<0.001
**Biochemical tests**			
**%**Anemia *<13.5* g/dL *[male]* *<12* g/dL *[female]*	11.1	29.6	0.063
%Iron deficiency *<49* µg/dL *[male]* *<37* µg/dL *[female]*	0	3.7	1.000
%Low ferritin levels (<30 ng/mL)	7.4	11.1	1.000
%Low transferrin saturation *(<20%)*	48.1	33.3	0.289
%Folate deficiency *(<2.76* ng/mL*)*	0	0	NA ^2^
%Vitamin B12 deficiency *(<239* pg/mL*)*	0	3.7	1.000
%Vitamin D deficiency *(<20* ng/mL*)*	22.2	18.5	1.000
%Vitamin A deficiency *(<30* µg/dL*)*	-	15.4	-

Abbreviations: body mass index (BMI); waist circumference (WC); excess weight loss (EWL). ^1^ All data presented as median and interquartile range unless otherwise stated. ^2^ NR= unable to compute.

## Data Availability

The data supporting this study’s findings are available from the corresponding author (S.S.-D.) upon reasonable request.

## Supplementary Materials

The following supporting information can be downloaded at: https://www.mdpi.com/article/10.3390/nu16162596/s1, Table S1: Carotenoid levels before and 6 months post-OAGB (N = 27); Figure S1: Individual variations in absolute carotenoid levels at baseline (T0) and 6 months post-OAGB (T6) (N = 27).

## Abbreviations

Biliopancreatic diversion (BPD), body mass index (BMI), double-distilled water (DDW), excess weight loss (EWL), Food Frequency Questionnaire (FFQ), High-performance liquid chromatography (HPLC), interquartile range (IQR), metabolic bariatric surgery (MBS), one anastomosis gastric bypass (OAGB), Roux-en-Y gastric bypass (RYGB), total weight loss (TWL), waist circumference (WC).

## References

[1] Wharton S., Lau D.C., Vallis M., Sharma A.M., Biertho L., Campbell-Scherer D., Adamo K., Alberga A., Bell R., Boulé N. (2020). Obesity in adults: A clinical practice guideline. Can. Med. Assoc. J..

[2] Perdomo C.M., Cohen R.V., Sumithran P., Clément K., Frühbeck G. (2023). Contemporary medical, device, and surgical therapies for obesity in adults. Lancet.

[3] Mechanick J.I., Apovian C., Brethauer S., Garvey W.T., Joffe A.M., Kim J., Kushner R.F., Lindquist R., Pessah-Pollack R., Seger J. (2020). Clinical practice guidelines for the perioperative nutrition, metabolic, and nonsurgical support of patients undergoing bariatric procedures—2019 update: Cosponsored by American Association of Clinical Endocrinologists/American College of Endocrinology, The Obesity Society, American Society for Metabolic & Bariatric Surgery, Obesity Medicine Association, and American Society of Anesthesiologists. Surg. Obes. Relat. Dis..

[4] Piazza L., Ferrara F., Leanza S., Coco D., Sarvà S., Bellia A., Di Stefano C., Basile F., Biondi A. (2011). Laparoscopic mini-gastric bypass: Short-term single-institute experience. Updat. Surg..

[5] Rutledge R. (2001). The mini-gastric bypass: Experience with the first 1274 cases. Obes. Surg..

[6] Mahawar K.K., Borg C.-M., Kular K.S., Courtney M.J., Sillah K., Carr W.R.J., Jennings N., Madhok B., Singhal R., Small P.K. (2017). Understanding Objections to One Anastomosis (Mini) Gastric Bypass: A Survey of 417 Surgeons Not Performing this Procedure. Obes. Surg..

[7] Bariatric Surgery Registry of the Ministry of Health, 2020. The Ministry of Health Web Site. https://www.health.gov.il/PublicationsFiles/Bariatric_2020.pdf.

[8] Bariatric Surgery Registry of the Ministry of Health, 2021. The Ministry of Health Web Site. https://www.gov.il/BlobFolder/reports/bariatric-2021/he/files_publications_units_ICDC_Bariatric_2021.pdf.

[9] Angrisani L., Santonicola A., Iovino P., Palma R., Kow L., Prager G., Ramos A., Shikora S. (2024). IFSO Worldwide Survey 2020–2021: Current Trends for Bariatric and Metabolic Procedures. Obes. Surg..

[10] Sherf-Dagan S., Biton R., Ribeiro R., Kessler Y., Raziel A., Rossoni C., Kais H., Bragança R., Santos Z., Goitein D. (2023). Nutritional and Lifestyle Behaviors Reported Following One Anastomosis Gastric Bypass Based on a Multicenter Study. Nutrients.

[11] Bruzzi M., Rau C., Voron T., Guenzi M., Berger A., Chevallier J.-M. (2015). Single anastomosis or mini-gastric bypass: Long-term results and quality of life after a 5-year follow-up. Surg. Obes. Relat. Dis..

[12] Charalampos T., Maria N., Vrakopoulou V.G.Z., Tania T., Raptis D., George Z., Emmanouil L., Konstantinos A. (2019). Tailored One Anastomosis Gastric Bypass: 3-Year Outcomes of 94 Patients. Obes. Surg..

[13] Jedamzik J., Eilenberg M., Felsenreich D.M., Krebs M., Ranzenberger-Haider T., Langer F.B., Prager G. (2020). Impact of limb length on nutritional status in one-anastomosis gastric bypass: 3-year results. Surg. Obes. Relat. Dis..

[14] Kessler Y., Adelson D., Mardy-Tilbor L., Ben-Porat T., Szold A., Goitein D., Sakran N., Raziel A., Sherf-Dagan S. (2020). Nutritional status following One Anastomosis Gastric Bypass. Clin. Nutr..

[15] Liagre A., Debs T., Kassir R., Ledit A., Juglard G., du Rieu M.C., Lazzati A., Martini F., Petrucciani N. (2020). One Anastomosis Gastric Bypass with a Biliopancreatic Limb of 150 cm: Weight Loss, Nutritional Outcomes, Endoscopic Results, and Quality of Life at 8-Year Follow-Up. Obes. Surg..

[16] Omar I., Sam M.A., Pegler M.E., Pearson E.J.B., Boyle M., Mahawar K. (2021). Effect of One Anastomosis Gastric Bypass on Haematinics, Vitamin D and Parathyroid Hormone Levels: A Comparison Between 150 and 200 cm Bilio-Pancreatic Limbs. Obes. Surg..

[17] Komaei I., Sarra F., Lazzara C., Ammendola M., Memeo R., Sammarco G., Navarra G., Currò G. (2019). One Anastomosis Gastric Bypass–Mini Gastric Bypass with Tailored Biliopancreatic Limb Length Formula Relative to Small Bowel Length: Preliminary Results. Obes. Surg..

[18] Elgeidie A., El-Magd E.-S.A., Elghadban H., Abdelgawad M., Hamed H. (2020). Protein Energy Malnutrition After One-Anastomosis Gastric Bypass with a Biliopancreatic Limb ≤200 cm: A Case Series. J. Laparoendosc. Adv. Surg. Tech..

[19] Khalaj A., Motamedi M.A.K., Mousapour P., Valizadeh M., Barzin M. (2019). Protein-Calorie Malnutrition Requiring Revisional Surgery after One-Anastomosis-Mini-Gastric Bypass (OAGB-MGB): Case Series from the Tehran Obesity Treatment Study (TOTS). Obes. Surg..

[20] Tasdighi E., Barzin M., Mahawar K.K., Hosseinpanah F., Ebadinejad A., Taraghikhah N., Mansoori A., Khalaj A., Niroomand M., Valizadeh M. (2022). Effect of Biliopancreatic Limb Length on Weight Loss, Postoperative Complications, and Remission of Comorbidities in One Anastomosis Gastric Bypass: A Systematic Review and Meta-analysis. Obes. Surg..

[21] Gentileschi P., Siragusa L., Alicata F., Campanelli M., Bellantone C., Musca T., Bianciardi E., Arcudi C., Benavoli D., Sensi B. (2022). Nutritional Status after Roux-En-Y (Rygb) and One Anastomosis Gastric Bypass (Oagb) at 6-Month Follow-Up: A Comparative Study. Nutrients.

[22] Granado-Lorencio F., Simal-Antón A., Blanco-Navarro I., González-Dominguez T., Pérez-Sacristán B. (2011). Depletion of serum carotenoid and other fat-soluble vitamin concentrations following obesity surgery. Obes. Surg..

[23] Böhm V., Lietz G., Olmedilla-Alonso B., Phelan D., Reboul E., Bánati D., Borel P., Corte-Real J., de Lera A.R., Desmarchelier C. (2021). From carotenoid intake to carotenoid blood and tissue concentrations—Implications for dietary intake recommendations. Nutr. Rev..

[24] Saini R.K., Prasad P., Lokesh V., Shang X., Shin J., Keum Y.S., Lee J.-H. (2022). Carotenoids: Dietary Sources, Extraction, Encapsulation, Bioavailability, and Health Benefits-A Review of Recent Advancements. Antioxidants.

[25] Arscott S.A., Tanumihardjo S.A. (2013). Food Sources of Carotenoids. Carotenoids and Human Health.

[26] Harrison E.H. (2019). Mechanisms of Transport and Delivery of Vitamin A and Carotenoids to the Retinal Pigment Epithelium. Mol. Nutr. Food Res..

[27] Eroglu A., Al’abri I.S., Kopec R.E., Crook N., Bohn T. (2023). Carotenoids and Their Health Benefits as Derived via Their Interactions with Gut Microbiota. Adv. Nutr. Int. Rev. J..

[28] Martini D., Negrini L., Marino M., Riso P., Del Bo C., Porrini M. (2022). What Is the Current Direction of the Research on Carotenoids and Human Health? An Overview of Registered Clinical Trials. Nutrients.

[29] Rocha H.R., Coelho M.C., Gomes A.M., Pintado M.E. (2023). Carotenoids Diet: Digestion, Gut Microbiota Modulation, and Inflammatory Diseases. Nutrients.

[30] Aune D., Keum N., Giovannucci E., Fadnes L.T., Boffetta P., Greenwood D.C., Tonstad S., Vatten L.J., Riboli E., Norat T. (2018). Dietary intake and blood concentrations of antioxidants and the risk of cardiovascular disease, total cancer, and all-cause mortality: A systematic review and dose-response meta-analysis of prospective studies. Am. J. Clin. Nutr..

[31] Harari A., Coster A.C.F., Jenkins A., Xu A., Greenfield J.R., Harats D., Shaish A., Samocha-Bonet D. (2020). Obesity and Insulin Resistance Are Inversely Associated with Serum and Adipose Tissue Carotenoid Concentrations in Adults. J. Nutr..

[32] Peng C., Gao C., Lu D., Rosner B.A., Zeleznik O., Hankinson S.E., Kraft P., Eliassen A.H., Tamimi R.M. (2021). Circulating carotenoids and breast cancer among high-risk individuals. Am. J. Clin. Nutr..

[33] El-Sohemy A., Baylin A., Kabagambe E., Ascherio A., Spiegelman D., Campos H. (2002). Individual carotenoid concentrations in adipose tissue and plasma as biomarkers of dietary intake. Am. J. Clin. Nutr..

[34] Granado-Lorencio F., Herrero-Barbudo C., Olmedilla-Alonso B., Blanco-Navarro I., Pérez-Sacristán B. (2009). Hypocarotenemia after bariatric surgery: A preliminary study. Obes. Surg..

[35] Kaniel O., Sherf-Dagan S., Szold A., Langer P., Khalfin B., Kessler Y., Raziel A., Sakran N., Motro Y., Goitein D. (2022). The Effects of One Anastomosis Gastric Bypass Surgery on the Gastrointestinal Tract. Nutrients.

[36] Sherf-Dagan S., Goldenshluger A., Globus I., Schweiger C., Kessler Y., Sandbank G.K., Ben-Porat T., Sinai T. (2017). Nutritional Recommendations for Adult Bariatric Surgery Patients: Clinical Practice. Adv. Nutr. Int. Rev. J..

[37] Bariatric Surgery Criteria of the Ministry of Health The Ministry of Health Web Site. http://www.health.gov.il/hozer/mr33_2013.pdf.

[38] Brethauer S.A., Kim J., el Chaar M., Papasavas P., Eisenberg D., Rogers A., Ballem N., Kligman M., Kothari S. (2015). Standardized outcomes reporting in metabolic and bariatric surgery. Surg. Obes. Relat. Dis..

[39] Pinho C.P.S., Diniz A.d.S., de Arruda I.K.G., Leite A.P.D.L., Petribu M.d.M.V., Rodrigues I.G. (2018). Waist circumference measurement sites and their association with visceral and subcutaneous fat and cardiometabolic abnormalities. Arq. Bras. de Endocrinol. Metabol..

[40] Sherf-Dagan S., Zelber-Sagi S., Webb M., Keidar A., Raziel A., Sakran N., Goitein D., Shibolet O. (2016). Nutritional Status Prior to Laparoscopic Sleeve Gastrectomy Surgery. Obes. Surg..

[41] (2015). Tzameret—Israeli National Nutrient Database 2015.

[42] Benotti P.N., Wood G.C., Kaberi-Otarod J., Still C.D., Gerhard G.S., Bistrian B.R. (2020). New concepts in the diagnosis and management approach to iron deficiency in candidates for metabolic surgery: Should we change our practice?. Surg. Obes. Relat. Dis..

[43] Borel P., Moussa M., Reboul E., Lyan B., Defoort C., Vincent-Baudry S., Maillot M., Gastaldi M., Darmon M., Portugal H. (2007). Human plasma levels of vitamin E and carotenoids are associated with genetic polymorphisms in genes involved in lipid metabolism. J. Nutr..

[44] Yeum K.J., Booth S.L., Sadowski J., Liu C., Tang G., Krinsky N., Russell R.M. (1996). Human plasma carotenoid response to the ingestion of controlled diets high in fruits and vegetables. Am. J. Clin. Nutr..

[45] Kim H.-Y. (2015). Statistical notes for clinical researchers: Effect size. Restor. Dent. Endod..

[46] Ben-Porat T., Elazary R., Goldenshluger A., Sherf Dagan S., Mintz Y., Weiss R. (2017). Nutritional deficiencies four years after laparoscopic sleeve gastrectomy-are supplements required for a lifetime?. Surg. Obes. Relat. Dis..

[47] Omenn G.S., Goodman G.E., Thornquist M.D., Balmes J., Cullen M.R., Glass A., Keogh J.P., Meyskens F.L., Valanis B., Williams J.H. (1996). Effects of a combination of beta carotene and vitamin A on lung cancer and cardiovascular disease. N. Engl. J. Med..

[48] Alpha-Tocopherol Beta Carotene Cancer Prevention Study Group (1994). The effect of vitamin E and beta carotene on the incidence of lung cancer and other cancers in male smokers. N. Engl. J. Med..

[49] U S. Department of Agriculture, Agricultural Research Service, Beltsville Human Nutrition Research Center. Food Data Central..

[50] Bonet M.L., Canas J.A., Ribot J., Palou A. (2015). Carotenoids and their conversion products in the control of adipocyte function, adiposity and obesity. Arch. Biochem. Biophys..

