# Supplementary material for: Reduction in Serum Carotenoid Levels Following One Anastomosis Gastric Bypass
Source: Nutrients. 2024 Aug 7;16(16):2596. doi: 10.3390/nu16162596 (PMC11357438; doi:10.3390/nu16162596)
Supplement: Supplementary file 1 [file nutrients-16-02596-s001.zip › nutrients-3110373-supplementary.pdf]

Supplementary Table S1: Carotenoid levels before and 6 months post-OAGB (N=27)

**Carotenoid[1]**	**Baseline**	**6 months post-surgery**	**P value**
**Absolute carotenoid levels [Median (µg/l)]**			
Lutein	105.0 (84.3, 138.9)	36.2 (27.8, 51.0)	**<0.001**
Zeaxanthin	11.0 (7.8, 15.7)	4.8 (3.5, 6.6)	**<0.001**
Phytofluene	29.9 (13.0, 44.5)	10.5 (7.2, 19.8)	**<0.001**
Alpha-carotene	40.6 (28.2, 62.4)	17.2 (11.4, 21.4)	**<0.001**
Beta-carotene	143.0 (104.0, 191.5)	64.0 (43.4, 88.4)	**<0.001**
Zeta-carotene	43.9 (31.7, 61.8)	19.1 (11.9, 32.7)	**<0.001**
Lycopene	236.6 (199.4, 309.6)	64.8 (50.9, 91.3)	**<0.001**
Total carotenoids	618.4 (528.9, 832.2)	216.3 (183.5, 303.6)	**<0.001**
**Adjusted carotenoid to total cholesterol levels [Median (µg/mg)]**			
Lutein	0.058 (0.042, 0.079)	0.022 (0.016, 0.032)	**<0.001**
Zeaxanthin	0.006 (0.004, 0.008)	0.003 (0.002, 0.005)	**<0.001**
Phytofluene	0.013 (0.007, 0.026)	0.007 (0.005, 0.011)	**0.003**
Alpha-carotene	0.020 (0.015, 0.032)	0.010 (0.007, 0.015)	**<0.001**
Beta-carotene	0.070 (0.053, 0.111)	0.045 (0.027, 0.054)	**<0.001**
Zeta-carotene	0.023 (0.019, 0.033)	0.014 (0.009, 0.020)	**<0.001**
Lycopene	0.120 (0.097, 0.186)	0.043 (0.027, 0.055)	**<0.001**
Total carotenoids	0.366 (0.264, 0.459)	0.137 (0.111, 0.196)	**<0.001**

Supplementary Figure S1: Individual variations in absolute carotenoid levels at baseline (T0) and 6 months post-OAGB (T6) (N=27)

**Presented as estimated marginal means

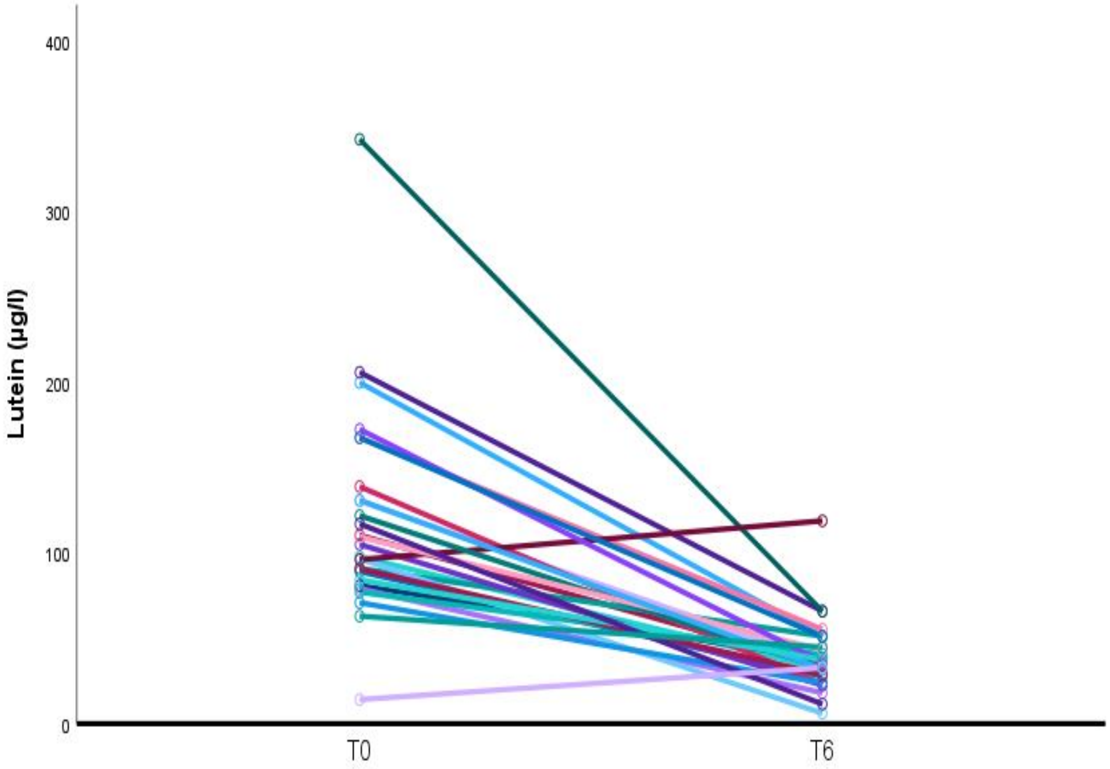

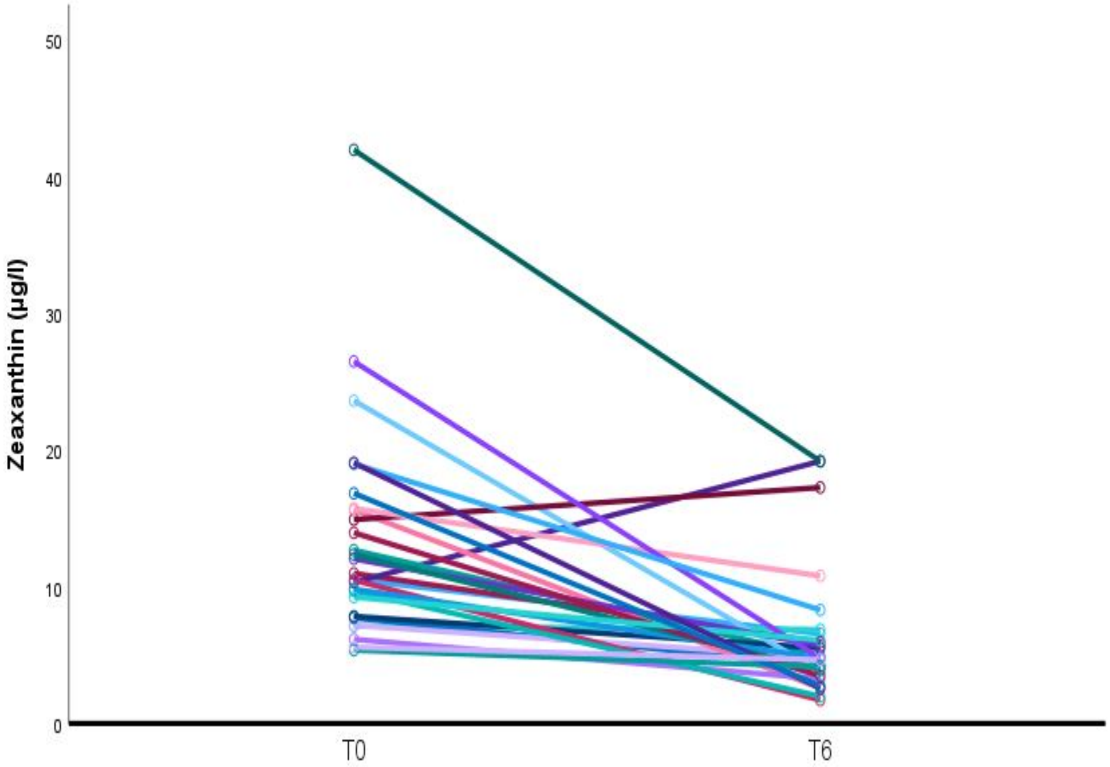

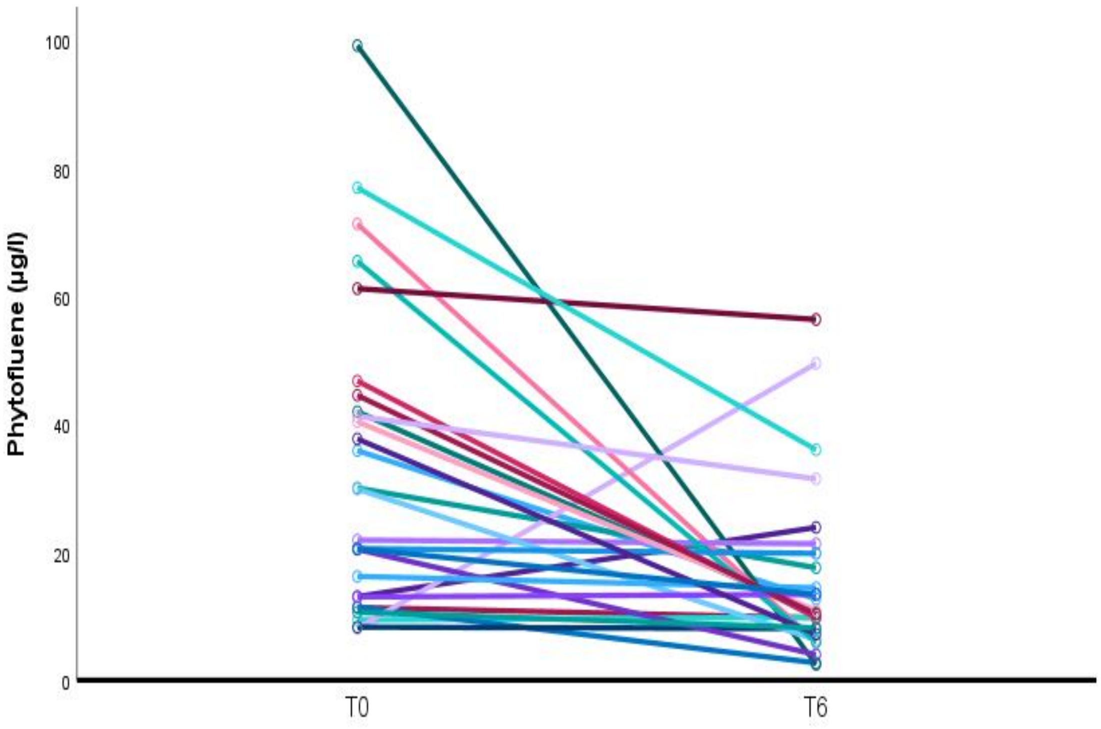
Phytofluene (µg/l)
100
80
60
40
20
0
T0
T6

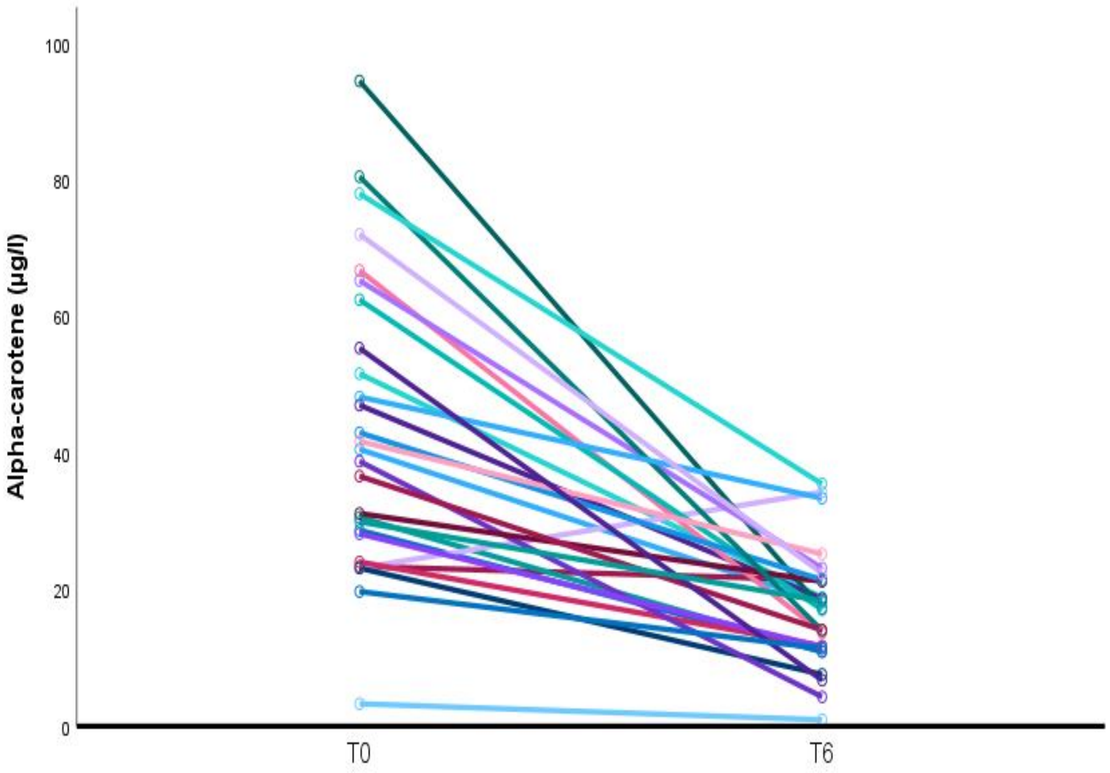
Alpha-carotene (µg/l)
100
80
60
40
20
0
T0
T6

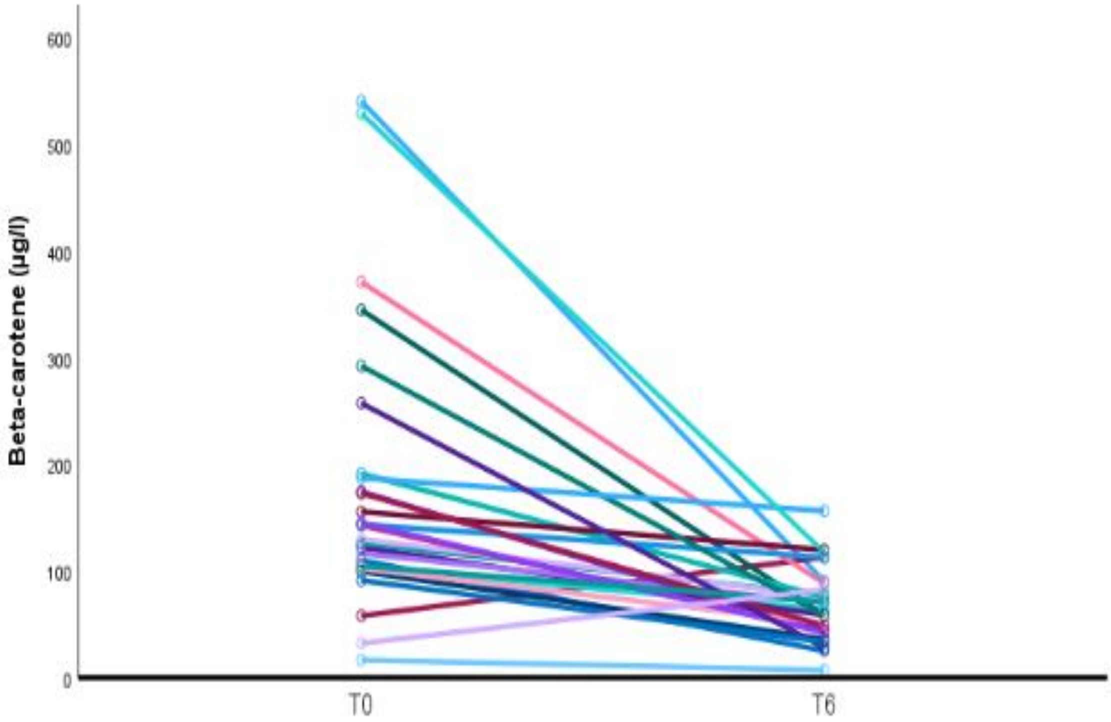
Beta-carotene (µg/l)
600
500
400
300
200
100
0
T0
T6

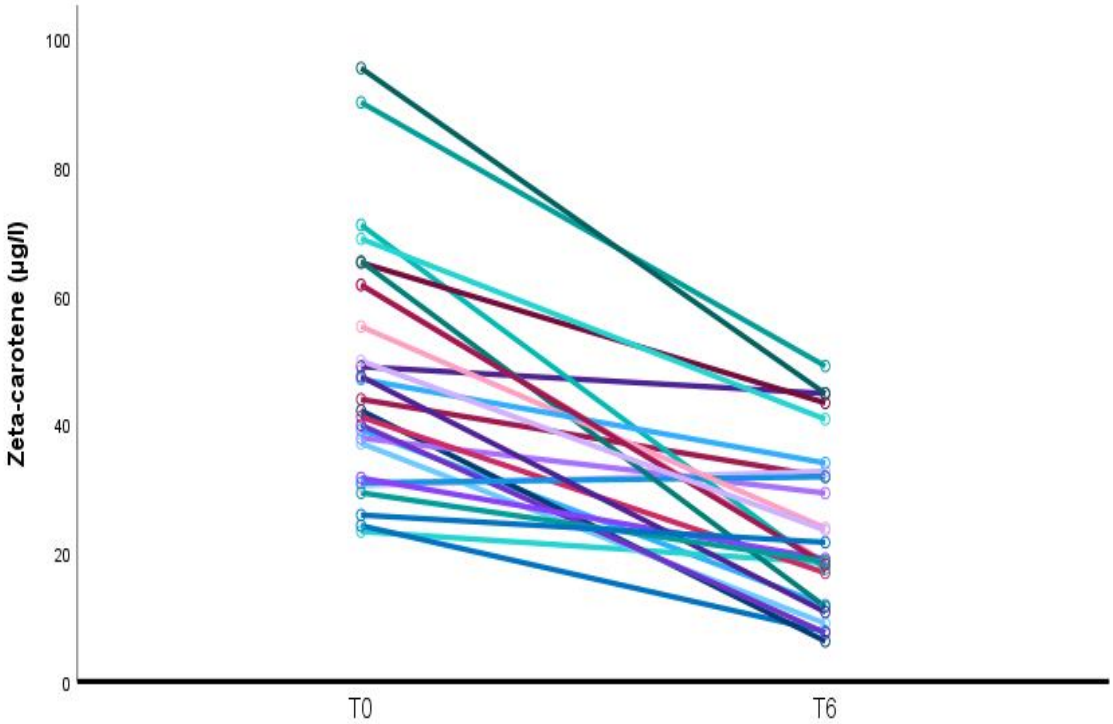
Zeta-carotene (µg/l)
100
80
60
40
20
0
T0
T6

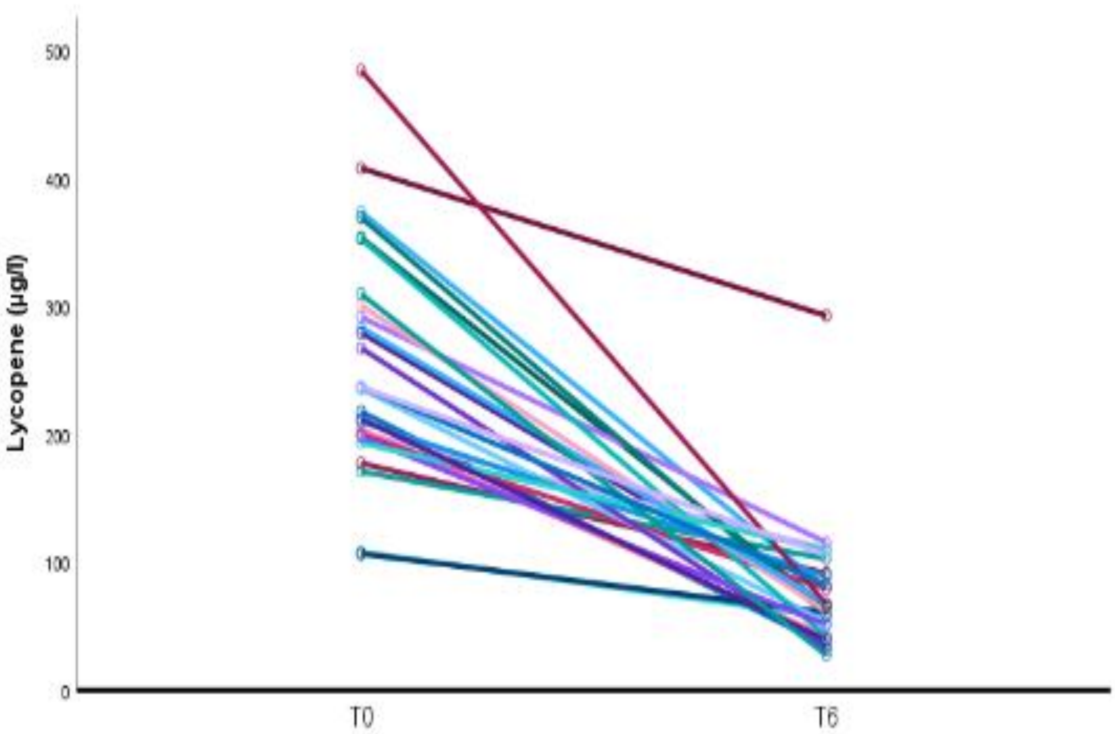
Lycopene (µg/l)
500
400
300
200
100
0
T0
T6
